# Murine Typhus from Vietnam, Imported into Japan

**DOI:** 10.3201/eid1209.060071

**Published:** 2006-09

**Authors:** Momoyo Azuma, Yasuhiko Nishioka, Motohiko Ogawa, Tomohiko Takasaki, Saburo Sone, Tsuneo Uchiyama

**Affiliations:** * University of Tokushima Graduate School, Tokushima, Japan;; † National Institute for Infectious Diseases, Tokyo, Japan

**Keywords:** cross-absorption, fluorescent antibody technique, Japan, Rickettsia prowazekii, Rickettsia typhi, typhus, epidemic louse-borne, typhus, endemic flea-borne, Vietnam, letter

**To the Editor:** In Vietnam, many febrile diseases such as malaria, dengue fever, Japanese encephalitis, scrub typhus, and more recently, severe acute respiratory syndrome (SARS) and avian influenza have been reported. Murine typhus cases were also reported during and before the 1960s but not thereafter (*1**–**5*).

On May 3, 2003, a 54-year-old male resident of Tokushima, Japan, had onset of fever in the suburban town of Cu Chi, ≈60 km northwest of Ho Chi Minh City, Vietnam. Exanthema appeared on his trunk and limbs on May 7. He returned to Japan on May 9 and was admitted to Tokushima University Hospital on May 10. His body temperature was 39.0°C, and serum, C-reactive protein level was high (17.06 mg/dL) on admission (day 8 of illness). Unfortunately, the blood sample taken on that day was discarded. We then collected blood on days 10, 11, 12, 14, 17, and 24 of illness for diagnosis. Minocycline was administered on day 8 and resulted in a gradual decrease in fever and rash. Weil-Felix tests on day 12 showed the serum to be positive for *Proteus vulgaris* OX19 (titer 160); tests for *P. vulgaris* OX2 and OXK were negative (titer of 10 for both). We examined blood samples for possible diseases such as malaria, dengue fever, SARS, and rickettsioses. Giemsa-stained peripheral blood samples obtained on day 11 showed no malarial parasites. Results of immunoglobulin M (IgM)-capture ELISA of serum on days 10, 11, and 17 of illness were negative for dengue antibodies. Reverse transcription (RT)–PCR of the serum on day 11 was also negative. RT-PCRs of a pharyngeal swab and urine collected on day 11 were both negative for the SARS coronavirus. These specimens were also injected into Vero cells, and no cytopathic effects were generated. RT-PCR of these cultures was also negative for SARS coronavirus. Moreover, SARS antibodies were not found in serum samples on days 11 and 14 of illness. Serum was also tested for *Orientia tsutsugamushi* and *Coxiella burnttii* on day 12 to exclude scrub typhus and Q fever as diagnoses.

Indirect immunofluorescence tests for etiologic agents of spotted fever, murine typhus, and epidemic typhus were then performed with serum samples collected on days 10, 14, and 24. We used *Rickettsia typhi* and *R. prowazekii* as typhus group (TG) rickettsial antigens and *R. japonica* and *R. conorii* as spotted fever group (SFG) rickettsiae. IgM antibody was detected for these antigens, indicating that the disease was a primary infection of rickettsiae (Table). When TG and SFG rickettsioses were compared, TG rickettsiae represented markedly higher elevated titers than SFG rickettsiae, which excluded a diagnosis of SFG rickettsiosis. PCR for the TG rickettsial genome in the convalescent-phase serum on day 10 was negative.

**Table:** IFA titers of the patient sera and the cross-absorption test*

Day of illness	Immunoglobulin class	Antigen for absorption	Antigen for IFA titration			
			TG rickettsiae		SFG rickettsiae	
			*R. typhi†*	*R. prowazekii‡*	*R. japonica*§	*R. conorii*¶
10	IgG	(–)	320	320	<20	20
	IgM	(–)	160	40	20	20
14	IgG	(–)	1,280	640	<20	40
		*R. typhi*	<20	<20	<20	<20
		*R. prowazekii*	160	0	<20	<20
		(–)	640	320	80	80
	IgM	*R. typhi*	<20	<20	<20	<20
		*R. prowazekii*	160	<20	<20	<20
24	IgG	(–)	640	640	<20	40
	IgM	(–)	640	320	80	80

*IFA, indirect immunofluorescence assay; IgG, immunoglobulin G; TG, typhus group; SFG, spotted fever group. †Strain Wilmington. ‡Strain Breinl. §Strain YH. ¶Strain Malish 7.

To demonstrate more detailed antigenic reactivity, Western immunoblotting was performed with serum on day 14 (*6*). The serum reacted similarly to the ladderlike lipopolysaccharide (LPS) of *R. typhi* and *R. prowazekii*. As expected from the group-specific nature of rickettsial LPS, no reaction was demonstrated to LPS of SFG rickettsiae, *R. japonica* and *R. conorii*, although weak reactivity, mainly to the major outer member protein of SFG rickettiae, rOmpB, and molecules of smaller sizes was shown (*6**,**7*). As described previously, rOmpB has cross-reactive antigenicity between TG and SFG rickettsiae (*6*). Compared with the trace reaction to rOmpB of SFG rickettsiae, an extremely high level of reaction was demonstrated to rOmpB of TG rickettsiae. These results confirmed the disease to be a TG rickettsiosis.

To elucidate whether the disease was murine typhus or epidemic typhus, we conducted cross-absorption tests as described previously (*8**,**9*). Serum absorbed by *R. typhi* showed complete absorption, demonstrating no reaction to *R. typhi* or *R. prowazekii* (Table). However, the serum absorbed by *R. prowazekii* resulted in incomplete absorption, demonstrating no reactivity to *R. prowazekii* but some reactivity to *R. typhi*, which was left unabsorbed. Western immunoblotting with the serum absorbed by *R. prowazekii* showed reactivity only to the rOmpB of *R. typhi* but not to that of *R. prowazekii*. These results confirmed the diagnosis of murine typhus.

This is the first serodiagnosis of murine typhus in Vietnam since the 1960s (*1**–**5*). Since rats inhabit the area where the patient acquired the illness, murine typhus seems to have occurred sporadically or endemically but to have been undiagnosed since the 1960s, maybe because it was thought to have been eradicated and thus widely forgotten. This case was the first imported into Japan since the 1940s, when many Japanese soldiers and residents who returned from abroad had the disease.
